# Genome analysis of *Mycoplasma synoviae* strain MS-H, the most common *M. synoviae* strain with a worldwide distribution

**DOI:** 10.1186/s12864-018-4501-8

**Published:** 2018-02-02

**Authors:** Ling Zhu, Muhammad A. Shahid, John Markham, Glenn F. Browning, Amir H. Noormohammadi, Marc S. Marenda

**Affiliations:** 1 Asia-Pacific Centre for Animal Health, Melbourne Veterinary School, Faculty of Veterinary and Agricultural Sciences, The University of Melbourne, Werribee, VIC 3030 Australia; 2 Department of Pathobiology, Faculty of Veterinary Sciences, Bahauddin Zakariya University, Multan, Punjab 60800 Pakistan; 3 Department of Electrical and Electronic Engineering, The University of Melbourne, Parkville, VIC 3000 Australia; 4 Asia-Pacific Centre for Animal Health, Melbourne Veterinary School, Faculty of Veterinary and Agricultural Sciences, The University of Melbourne, Parkville, VIC 3010 Australia

**Keywords:** *Mycoplasma synoviae*, MS-H vaccine strain, Complete genome sequencing, Comparative genetic analysis, Large chromosomal inversion, Vaccine-specific genetic markers

## Abstract

**Background:**

The bacterial pathogen *Mycoplasma synoviae* can cause subclinical respiratory disease, synovitis, airsacculitis and reproductive tract disease in poultry and is a major cause of economic loss worldwide. The *M. synoviae* strain MS-H was developed by chemical mutagenesis of an Australian isolate and has been used as a live attenuated vaccine in many countries over the past two decades. As a result it may now be the most prevalent strain of *M. synoviae* globally. Differentiation of the MS-H vaccine from local field strains is important for epidemiological investigations and is often required for registration of the vaccine.

**Results:**

The complete genomic sequence of the MS-H strain was determined using a combination of Illumina and Nanopore methods and compared to WVU-1853, the *M. synoviae* type strain isolated in the USA 30 years before the parent strain of MS-H, and MS53, a more recent isolate from Brazil. The vaccine strain genome had a slightly larger number of pseudogenes than the two other strains and contained a unique 55 kb chromosomal inversion partially affecting a putative genomic island. Variations in gene content were also noted, including a deoxyribose-phosphate aldolase (*deoC*) fragment and an ATP-dependent DNA helicase gene found only in MS-H. Some of these sequences may have been acquired horizontally from other avian mycoplasma species.

**Conclusions:**

MS-H was somewhat more similar to WVU-1853 than to MS53. The genome sequence of MS-H will enable identification of vaccine-specific genetic markers for use as diagnostic and epidemiological tools to better control *M. synoviae*.

**Electronic supplementary material:**

The online version of this article (10.1186/s12864-018-4501-8) contains supplementary material, which is available to authorized users.

## Background

The avian pathogen *Mycoplasma synoviae* is a member of the class *Mollicutes*, a group of bacteria that are characterised by their very small size, lack of cell wall, complex nutritional requirements and ability to persist in their hosts and establish chronic infections [1]. *M. synoviae* strains appear to have varying tissue tropisms and virulence, although these characteristics may depend on the route of infection [2]. It causes subclinical upper respiratory tract infections in chickens, turkeys and other birds [3]. It can also disseminate further into the host, leading to synovitis or egg defects [4]. It is transmitted horizontally via direct contact, or vertically via fertile eggs [1]. Although *M. synoviae* is rarely associated with bird mortality, its impact on avian health is significant. As a result of the implementation of adequate control programs for *M. gallisepticum*, the other major mycoplasmal pathogen of poultry, *M. synoviae* may now be the most important bacterial cause of economic loss in the poultry industry [5]. Vaccination is commonly used to control *M. synoviae* infection in commercial flocks in many countries with significant commercial poultry industries. The temperature-sensitive strain MS-H was produced by *N*-nitro-*N’*methyl-*N*-nitrosoguanidine (NTG) chemical mutagenesis of an Australian field isolate, 86,079/7NS [6, 7]. MS-H does not grow at the core body temperature of birds, colonises only their upper respiratory tract and establishes solid protection against wild type *M. synoviae*. MS-H was first registered as a live attenuated vaccine in Australia in 1996 (Vaxsafe MS; Bioproperties Pty. Ltd., Ringwood, Victoria, Australia) and is now formally registered and used for vaccination of commercial poultry in 26 different countries across 6 continents. In addition, MS-H is used in several other countries where formal registration is not required (personal communications with Dr. Ross Henderson and Dr. Chris Morrow, Bioproperties Australia Pty. Ltd.). The international use of the MS-H vaccine suggests that it may now be the most common strain of *M. synoviae* globally and highlights the importance of assays developed to differentiate local endemic strains from the vaccine [8]. However, little information is available about the genetic relatedness of MS-H and other *M. synoviae* field strains found in poultry. Moderately virulent *M. synoviae* strains have been isolated from flocks previously vaccinated with MS-H [9], raising the question of the origin of these organisms and prompting interest in the sequence of the MS-H genome. Such knowledge would help to define molecular markers for tracking MS-H in vaccinated flocks and assessing any variation in the level of cross-protection against local strains of *M. synoviae*. The sequences of the *M. synoviae* field strain MS53, isolated around 2003 from a broiler flock in Brazil [10], and the type strain WVU-1853, isolated in 1957 from a hock joint of chicken in the USA [11], are the only two complete genomes determined thus far for this species. This is possibly due to difficulties experienced in assembling next generation sequencing (NGS) data for *M. synoviae*, which contains large, low complexity, repeat-rich regions. Here, MS-H was completely sequenced by combining short, accurate Illumina sequence data with long reads generated using the Oxford Nanopore technology. This genome sequence was compared to the complete genome sequences of strains WVU-1853 or MS53 with the aim of identifying features unique to MS-H, WVU-1853 or MS53, and assessing the degree of overall similarity between the three genomes and identifying MS-H specific features that could be targeted to develop genotyping assays and differentiate the vaccine from field strains, enabling improved assessment of disease control strategies.

## Methods

### Preparation of genomic DNA and sequencing

*M. synoviae* strain MS-H was inoculated into mycoplasma culture medium containing 10% swine serum and 0.01% (*w*/*v*) nicotinamide adenine dinucleotide [12] and grown until late logarithmic phase (pH of approximately 6.8) at 37 °C in a 50 mL final volume. Cells were collected by centrifugation at 10000×g for 30 min. Genomic DNA was prepared by proteinase K digestion of the pellet, phenol-chloroform extraction and ethanol precipitation [13, 14]. An Illumina paired-end (300 bp insert size) genomic DNA library was prepared and sequenced by the Micromon DNA Sequencing Facility (Monash University, Australia) using a Genome Analyzer IIx system. An Oxford Nanopore long read genomic DNA library was prepared with the sequencing kit SQK-NSK007 (Oxford Nanopore Technologies, Oxford, OX4 4GA, UK) according to the manufacturer’s instructions using a mixture of 1.2 μg of genomic DNA sheared using a Covaris-g TUBE and 0.8 μg of unsheared genomic DNA. Sequencing data were generated on a MinION MK-I device fitted with a FLO-MIN104 flowcell (R9 chemistry) and processed using the cloud-based Metrichor workflow 2D base caller RNN SQK-NSK_007 rev 1.107. Oligonucleotides (Geneworks, Australia) were designed using Primer3 version 2.3.4 and PCR amplicons were sequenced using the Sanger method at the Micromon DNA Sequencing Facility (Monash University, Melbourne, Australia).

### De novo sequence assembly, gene prediction and annotation of MS-H

Paired-end Illumina reads were filtered to select those with quality values above 20 and a minimal read length of 91 after trimming of adapter sequences. The quality of the filtered reads was confirmed with the FastQC [15]. The Illumina reads were de novo assembled using Velvet [16] (version 1.2.10) using k-mer values of 81 and 91 and coverage cut-off values of 5, 10, 20, 50, 100 and 200. Nanopore 2D reads with lengths > 2500 bp were extracted from the set of fast5 files returned in the “pass” folder by the Metrichor basecaller and converted into .fasta format using Poretools [17]. The nanopore reads were de novo assembled with Canu 1.2 [18] using the parameters genomesize = 0.9 m and errorRate = 0.1. The Illumina read datasets were mapped against the Nanopore assembly and the consensus sequence extracted using the Geneious version 7.0.6 sequence manipulation suite. Automatic annotation of the MS-H sequence was performed on the RAST webserver with default parameters for mycoplasmas [19]. Provisional locus tag numbers were assigned and the corresponding nucleotide positions are listed in Additional file 1: Table S1.

### Genome comparison and analysis

To ensure consistency in the analysis, the genome sequences of *M. synoviae* strains MS53 and WVU-1853 (GenBank accession numbers AE017245 and CP011096) were re-annotated using the RAST website with default parameters as above. Genome characteristics of each *M. synoviae* strain were analysed using Artemis [20]. Whole genome sequence alignments were performed using the Mauve Aligner and LASTZ tools in Geneious version 7.0.6. Pairwise alignments of nucleotide and amino acid sequences of individual genes were performed using the generic Geneious alignment tool with the default parameters. The search for pathogenicity genomic islands was conducted using the IslandViewer3 server (http://www.pathogenomics.sfu.ca/islandviewer) and the SIGI-HMM and IslandPath-DIMOB methods [21]. The PHAge Search Tool (PHAST) (http://phast.wishartlab.com) was used to detect complete and/or incomplete prophage sequences [22]. Clustered regularly interspersed short palindromic repeats (CRISPR) sequences were detected using CRISPRfinder (http://crispr.i2bc.paris-saclay.fr) [23]. Insertion sequences (IS) were analysed using the ISFinder tool (http://www-is.biotoul.fr) [24].

## Results and Discussion

### Assembling MS-H genome sequence and resolving the highly repeated *vlhA* region

Extensive assembly attempts using Velvet with a set of 30,345,486 MS-H Illumina paired-end reads with various K-mer and coverage cutoff values produced 659 contigs with sizes ranging from 181 bp to 134,673 bp. These contigs were aligned to the strain MS53 genome to obtain assembly parameters. De novo assembly of the MS-H genome was then attempted. However, many genomic regions could not be assembled solely from the Illumina datasets. Sanger sequencing of 28 PCR products and scaffolding of the Velvet data produced a 766,314 bp contig, representing an almost complete genome, but failed to assemble the *vlhA* pseudogene region, a 50 kb locus containing a cluster of highly repeated sequences. To resolve the *vlhA* region and complete the MS-H genome, a set of 29,015 Nanopore reads containing a total of 209,108,300 nt were independently assembled and circularised into a single 810,924 bp contig. Because Nanopore sequences are error-prone, the Illumina reads were then mapped against the contig, providing an average coverage depth of 3661 ± 829, and the consensus sequence was then extracted from the aligned reads. This approach generated a fully assembled genome encompassing the entire *vlhA* cluster and all the other regions that were difficult to assemble solely from the Illumina reads. The final MS-H genome was then verified by aligning the previously obtained Velvet contigs and Sanger sequenced PCR products, where available, with the new assembly. All nucleotide discrepancies between the two datasets were resolved manually by inspecting the alignments of Illumina reads to verify the quality of the consensus. The results from the Illumina/Nanopore hybrid approach was found to be accurate in all cases, except for a dinucleotide repeat sequence, (CT)_15_, which was correctly re-interpreted as (CT)_13_. These results demonstrate that a strategy combining error-prone but long single-molecule reads (Nanopore) with accurate short next generation sequencing data (Illumina) can generate a high quality, complete mycoplasma genome sequence. This approach is particularly well adapted to *Mollicutes*, which often contain repeated regions that are difficult to assemble. As expected, the MS-H-specific mutation in the *obg* gene [25], previously reported as a marker of the vaccine strain, was identified. The whole-genome sequence of MS-H has been deposited in the National Centre for Biotechnology Information (NCBI) under accession number CP021129.

### General comparisons of *M. synoviae* genomes and identification of a large chromosomal inversion in MS-H

**Fig. 1:**
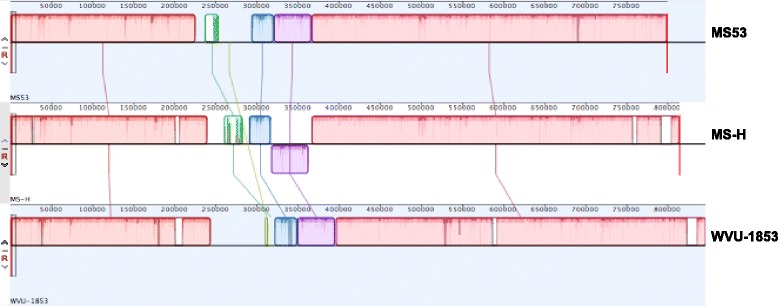
MAUVE alignment analysis of *M. synoviae* genomes, using MS-H as a reference. Similarly colored blocks with connecting lines represent homologous regions. A block below the centre line indicates a region that aligned in reverse complement (inverse) orientation

**Fig. 2:**
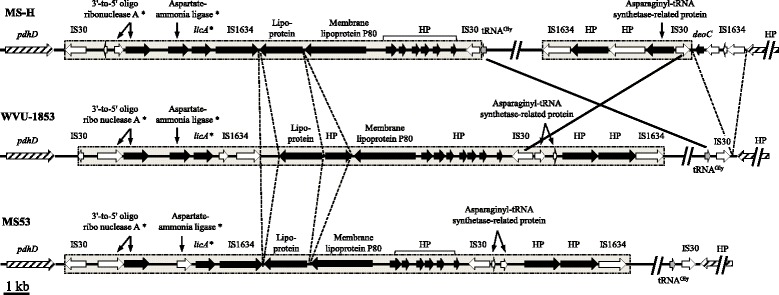
Comparative structural analysis of the genomic island (GI) in MS-H, WVU-1853 and MS53. The grey boxes with dotted lines indicate genes identified as part of the genomic island. Hatched arrows indicate genes flanking the GI. The inverted region in MS-H relative to MS53 and WVU-1853 is indicated by crossed solid lines. Inserted/deleted sequences are indicated by dashed lines. Genes with a complete predicted CDS are depicted in black; pseudogenes are depicted in white; tRNA genes are depicted in grey. HP: hypothetical protein. The asterisks indicate sequences duplicated elsewhere in the host genome

The general features of the MS-H, MS53 and WVU-1853 genomes are listed in Table 1. The *M. synoviae* MS-H genome was 818,848 bp long with an overall GC content of 28.2%. The DNA-DNA sequence identities between MS-H and WVU-1853 and between MS-H and MS53, (excluding the *vlhA* locus region) were 92.1% and 91.3% respectively. All 3 strains had similar average gene lengths, ranging from 992 to 1002 bp, average gene densities of approximately 90%, and numbers of putatively encoded proteins, ranging from 723 to 764, of which 541 to 573 had predicted functions. In each strain, 34 tRNAs and 7 rRNAs, consisting of three copies of 5S, two copies of 23S, and two copies of the 16S rRNA subunits, were identified. The strains had most of their genes or gene products in common, sharing 92.5% - 100% nucleotide sequence identities and 88.0% - 100% amino acid sequence identities. Comparisons of open reading frames (ORFs) revealed that 5 of the MS-H, 7 of the WVU-1853 and 3 of the MS53 ORFs have insertions or deletions in multiples of 3 bp, resulting in slightly longer or shorter proteins without frameshifts (Table 2). Analysis of the genomes of the 3 strains using MAUVE revealed that their chromosomes were collinear, with the remarkable exception of a unique 55 kb inversion in MS-H (Fig. 1) (Additional file 1: Table S1). Large chromosomal inversions have not been reported before in *M. synoviae*, but have been seen occasionally in other mollicutes, including *M. hyopneumoniae* [10], and *M. bovis* [26]. The molecular mechanism underlying this inversion, which is delimited by a tRNA^Gly^ and an IS30 (Fig. 2), is unclear. It has been proposed that the partial lack of DNA replication and repair functions in *Mycoplasma* species could prevent the formation of large genomic inversions [27]. The NTG-mutagenesis used to create the MS-H vaccine generates point mutations and is therefore unlikely to have caused this rearrangement. Whether this genomic inversion occurred naturally in the lineage of MS-H and is a common feature amongst Australian field strains, or was induced by mutagenesis will require further genomic sequencing from the parental strain of the vaccine, namely 86,079/7NS. Repeated sequences can contribute to chromosomal rearrangements, including inversions [28, 29]. The genomic structures of *Mycoplasma bovis* and *Mycoplasma agalactiae* have high syntenies except for a 142 kb inversion, which may be related to an IS*Mbov1* element adjacent to this region in *M. bovis* [26]. A similar mechanism may have generated the chromosomal rearrangement in MS-H. Apart from the inversion, the genomic organisation of MS-H was generally similar to those of MS53 and WVU-1853.

**Table 1:** General characteristic of the genomes of 3 *M. synoviae* strains MS-H, MS53 and WVU-1853

Characteristic	MS-H	MS53	WVU-1853
Total length (base pairs)	818,848	799,476	846,495
G + C content (%)	28.2	28.5	28.3
Gene density (%)	90.0	90.4	90.2
Average gene length (base pairs)	1002	994	992
No. features (genes)	775	764	805
No. coding sequences	734	723	764
No. CDS with predicted function	541	543	573
No. rRNAs			
16S	2	2	2
23S	2	2	2
5S	3	3	3
No. of tRNAs	34	34	34

**Table 2:** Unique amino acid deletions/insertions in *M. synoviae* strains MS-H, MS53 and WVU-1853

Features (Locus tag)	MS-H	MS53	WVU-1853
Cell division protein FtsZ (MS53_0340)		− 7	
Conserved hypothetical protein (MS53_0547)		− 4	
Conserved hypothetical protein (MS53_0590)		− 10	
Hypothetical protein (VY93_01870)			− 4
Hypothetical protein (VY93_02525)			+ 3
Hypothetical protein (VY93_02550)			− 29
Peptidase C1 (VY93_03235)			− 13
Signal peptidase I (VY93_00225)			− 6
PTS sugar transporter subunit IIABC (VY93_00785)			+ 8
ABC transporter ATP-binding protein (VY93_03350)			− 2
Choline kinase family (MSH_00560)	− 5		
Hypothetical protein (MSH_02310)	− 3		
Hypothetical protein (MSH_04020)	− 5		
Siderophore-mediated iron transport protein (MSH_05040)	− 20		
Hypothetical protein (MSH_05940)	+ 4		

“+” indicates amino acid insertion into the protein and “-” indicates amino acid deletion from the protein, relative to the other two strains. Nucleotide positions corresponding to the locus tags of MS-H are listed in Additional file 1: Table S1

### Large mobile genetic elements

Several large mobile genetic elements were identified in all three genomes. Two distinct clusters of phage-related sequences were found in MS-H, MS53 and WVU-1853. These two clusters were similarly organised across the strains and were located at nucleotide positions 446,358–459,743 and 126,056–134,674 in MS-H, 442,958–456,345 and 123,762–132,823 in MS53, and 472,072–485,459 and 124,910–133,971 in WVU-1853. Their GC contents were similar (28.72 ± 0.11% for the first cluster and 29.24 ± 0.05% for the second cluster) and slightly higher than the average GC content of the host genomes. In contrast, a third region, with significantly lower GC content (22.2%–24.2%) was predicted by IslandViewer3 analysis to be a putative genomic island (GI) in MS53 (nucleotide positions 302,828–327,410) and WVU-1853 (nucleotide positions 329,954–356,584). In MS-H, a related region was identified by sequence alignment with the two other strains; this region was adjacent to, and partially affected by, the 55 kb [tRNA^Gly^ - IS30] genomic inversion (Fig. 2) on its right side. Specifically, the left portion of the GI was located at nucleotide positions 303,839–321,538 while the right portion was found at 360049–367358 as a result of the genomic inversion. The GI encoded lipoproteins, hypothetical proteins and transposases of the IS30 and IS1634 families. Three coding sequences (CDSs) adjacent to the GI, encoding an oligoribonuclease A, an aspartate-ammonia ligase and a lichenan-specific IIA (LicA) component of a phosphotransferase system (PTS), were also found at another chromosomal locus. In the GI, these CDSs were flanked by an IS30 and an IS1634 (Fig. 2). In MS53, the GI-encoded aspartate-ammonia ligase was present as a pseudogene, while the other chromosomal copy was intact. In all 3 strains, the much lower GC content of the GI suggests that it was horizontally acquired by *M. synoviae* from another organism.

### Strain-variable sequences in *M. synoviae* genomes and vaccine-specific genetic markers

**Fig. 3:**
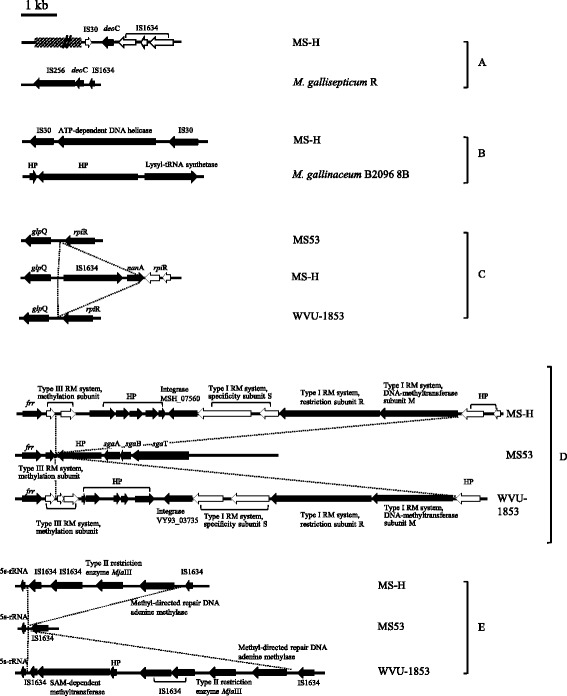
**a** Location of the *deoC* gene in *M. gallisepticum* corresponding to the *deoC* fragment found in MS-H. **b** Locations of the ATP-dependent DNA helicase genes in MS-H and *M*. *gallinaceum* strain B2096 8B. **c**, **d**, **e** Comparison of unique genomic loci identified in *Mycoplasma synoviae* strains MS-H, WVU-1853 and MS53. The inserted/deleted sequences are indicated by dotted lines. Genes with complete predicted CDSs are depicted in black; pseudogenes are depicted in white. HP: hypothetical protein

Comparison of the gene repertoires of the three strains (excluding the *vlhA* locus, which has complex patterns of variability within *M. synoviae*) revealed that 18 predicted gene products were variably distributed amongst the 3 strains (Table 3). These genes were either present in only one of the strains or present in two strains, but absent from the third. Strain-variable sequences are of particular interest because they could be used as molecular markers for differentiating the MS-H vaccine from field strains and understanding the recent evolution of *M. synoviae* and other avian mollicutes. Remarkably, most of the loci containing strain-variable genes were associated with, and often flanked by, IS elements. The comparative organisation of the major strain-variable loci is illustrated in Fig. 3. Only a few of the putative gene products from these loci had a predicted function at the time of analysis. They include a deoxyribose-phosphate aldolase (*deoC*) fragment (MSH_03520) (Additional file 2: Table S2), an ATP-dependent DNA helicase (MSH_07190), an N-acetylneuraminate lyase (*nanA*) variant (MSH_00300), an integrase (MSH_07560, VY93_03735), a DNA methylase (MSH_02150, VY93_01015/VY93_02585), and several type I, II and III related restriction-modification proteins. The *deoC* fragment and the ATP-dependent DNA helicase sequences were located at two distinct chromosomal sites in the MS-H genome, and were absent in WVU-1853 and MS53 (Fig. 3a and b). While a complete copy of *nanA* was found in MS-H, WVU-1853 and MS53 at a conserved locus, a second truncated sequence variant lacking the first 134 nt and adjacent to an IS1634 family transposase gene, was located between the conserved *glpQ* and *rpiR* genes in MS-H only (Fig. 3c). NanA is involved in sialic acid scavenging and degradation and is proposed to be associated to virulence in *M. synoviae* [30, 31]. Whether the *nanA* variant found in MS-H influences the sialic acid metabolism of the strain remains to be explored. A locus containing an integrase gene, several of hypothetical protein CDSs and sequences related to type I and type III restriction-modification systems were found in MS-H and WVU-1853 but not in MS53 (Fig. 3d). The type I and type III restriction-modification related sequences were located at both ends of the locus, in opposite orientations. These sequences were pseudogenes (see below) and had similarities with CDSs for two restriction S subunits, one restriction R subunit and one DNA-methyltransferase M subunit for the type I system, and two restriction-modification system methylation subunits for the type III system. Genes encoding type I restriction modification (RM) systems are known to be unstable and to display allelic variability [32]. Accordingly, no type I RM system was found in MS53 and significant sequence variations were seen between the specificity subunits; MS-H and WVU-1853 had four type III RM system methylation subunits, while only one was seen in MS53. Moreover, a locus encoding a type II restriction enzyme homologous to *MjaIII* (prototype *MboI*) and a methyl-directed repair DNA adenine methylase, flanked by IS1634 copies was detected in MS-H and WVU-1853, but not in MS53 (Fig. 3e). Overall, the analysis of the strain-variable gene repertoires indicated that strains MS-H and WVU-1853, which have distinct geographical and historical origins, were more closely related to each other than strain MS53, which was isolated from the same continent as WVU-1853. Whether these findings are representative of strain diversity in North and South America compared to Australia remains to be explored.

**Table 3:** Summary of variable genes across *M. synoviae* strains MS-H, MS53 and WVU-1853

Gene product	ORF (locus tag^a^)		
	MS-H	MS53	WVU-1853
N-acetylneuraminate lyase variant	MSH_00300	–	–
Transcriptional regulator	MSH_00310^b^ MSH_00330^b^	MS53_0024	VY93_00145
Translation elongation factor G	MSH_00500	MS53_0039^b^	VY93_00220^b^
Multiple sugar ABC transporter, ATP-binding protein	MSH_01200^c^	MS53_0102	VY93_00560
Hypothetical protein	MSH_01840^c^	–	VY93_00865
Hypothetical protein	MSH_01960	–	VY93_00910
Hypothetical protein	MSH_01930 MSH_01970	–	VY93_00915
Type II restriction enzyme *MjaIII*	MSH_02140	–	VY93_01010 VY93_02580
Methyl-directed repair DNA adenine methylase	MSH_02150	–	VY93_01015 VY93_02585
3′-to-5′ oligoribonuclease A	MSH_02940^b^ MSH_02950^b^ MSH_02960^b^	MS53_0278^b^ MS53_0279^b^	VY93_01505^b^ VY93_01510^b^
Aspartate-ammonia ligase	MSH_02990	MS53_0281^b^	VY93_01515
Hypothetical protein	MSH_03480^b^ MSH_03490^b^	MS53_0293	VY93_01595^b^
Asparaginyl-tRNA synthetase-related protein	MSH_03500	MS53_0291^b^ MS53_0700^b^	VY93_01590^b^
Deoxyribose-phosphate aldolase	MSH_03520	–	–
Oligopeptide transport ATP-binding protein OppF	MSH_03740^b^ MSH_03750^b^	MS53_0345	VY93_01875
Lipoprotein	MSH_03790	MS53_0349	VY93_01895^b^ VY93_01900^b^
Hypothetical protein	MSH_04620	MS53_0430^b^	VY93_02305
Hypothetical protein	MSH_04790^b^	MS53_0442	
Hypothetical protein	MSH_04880		VY93_02420
Hypothetical protein	MSH_04960	MS53_0458^b^ MS53_0459^b^	VY93_02465
Hypothetical protein	MSH_05850^b^ MSH_05860^b^	MS53_0713^b^ MS53_0540^b^	VY93_02935^b^
CRISPR-associated protein, Csn1	MSH_06430^b^ MSH_06440^b^	MS53_0582	VY93_03200^b^ VY93_03205^b^
IS30 family transposase	MSH_07180 MSH_07210	–	VY93_00200 VY93_00900 VY93_01540 VY93_02375
ATP-dependent DNA helicase	MSH_07190	–	–
Hypothetical protein	MSH_07360^b^	MS53_0668	VY93_03635
Hypothetical protein	MSH_07370^b^	MS53_0669^b^	VY93_03640
ABC transporter ATP-binding and permease protein	MSH_07380	MS53_0670^b^	VY93_03645^b^
Hypothetical protein	MSH_07610^c^	MS53_0680	VY93_03760^b^
PTS system enzyme IIB component	MSH_07640^b^	MS53_0682	VY93_03770
Ascorbate-specific PTS system, EIIC component	MSH_07650^b^ MSH_07660^b^ MSH_07670^b^ MSH_07680^b^	MS53_0683	VY93_03775^b^
Type III restriction-modification system methylation subunit	MSH_07490^b^ MSH_07500^b^	–	VY93_03700^b^ VY93_03705^b^
Hypothetical protein	MSH_07510	–	VY93_03710^b^
Hypothetical protein	MSH_07520	–	VY93_03720^b^
Hypothetical protein	MSH_07530	–	VY93_03725^b^
Hypothetical protein	MSH_07540 ^b^	–	VY93_03730
Integrase	MSH_07560	–	VY93_03735
Type I restriction-modification system, specificity subunit S	MSH_07570^b^ MSH_07580^b^	–	VY93_03740^b^ VY93_03745^b^
Type I restriction-modification system, restriction subunit R	MSH_07590	–	VY93_03750
Type I restriction-modification system, DNA-methyltransferase subunit M	MSH_07600	–	VY93_03755

^a^Pseudogenes that have lost more than 30% of their full length; nucleotide positions corresponding to the locus tags of MS-H are listed in Additional file 1: Table S1. IS elements and the *vlhA* locus were not included in pseudogenisation analysis. ^b^Pseudogenes that result from a frameshift mutation. ^c^ Pseudogenes that result from acquisition of an internal stop codon. “−” indicates the absence of the gene in a strain. Genes without a specified locus tag are not indicated in this table

### Pseudogenisation of transport systems and their potential impact on attenuation of virulence

**Fig. 4:**
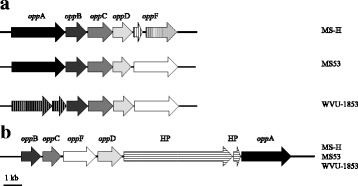
Organisation of the *opp* operons in MS-H, MS53 and WVU-1853. **a** Comparison of operon I in strains MS-H, MS53 and WVU-1853; (**b**) organisation of operon II in all three strains. Solid arrows indicate functional genes, while vertically hatched arrows indicate pseudogenes. Horizontally hatched arrows indicate hypothetical proteins (HP)

Excluding the IS elements and the *vlhA* locus, a total of 27 pseudogenes were identified in MS-H, WVU-1853 and MS53, with some differences in pseudogene repertoires between strains (Table 3). MS-H, WVU-1853 and MS53 had 17, 15 and 9 pseudogenes, respectively. Of these pseudogenes, 8 were unique to MS-H, 4 to WVU-1853 and 3 to MS53. Only 3 of the 27 were caused by a point mutation creating a TAA stop codon within the same frame. The majority of the pseudogenes were the result of single nucleotide insertions or deletions resulting in frameshifts. Overall, the numbers of pseudogenes were relatively low and similar across all strains, albeit slightly higher in MS-H. Active pseudogenisation in a live vaccine could lower its immunogenic capacity and efficacy by disrupting the expression of protective antigens. Based on our analysis, pseudogenisation does not seem to be significantly more prevalent in MS-H, and is unlikely to affect its repertoire of expressed antigens. The potential impact of pseudogenisation on the attenuation of virulence in MS-H was also considered. A remarkable example of pseudogenisation was found in the oligopeptide permease (Opp) system. Multiple copies of *opp* operons are commonly seen in both Gram-negative and Gram-positive bacteria, including *M. gallisepticum*, and can play a role in virulence [33–36]. The Opp systems comprise an extracellular substrate binding protein OppA, two transmembrane proteins OppB and OppC, which form the pore, and two cytoplasmic ATPases OppD and OppF, which provide the energy for peptide translocation [37, 38]. As in most mycoplasmas, all 3 *M. synoviae* strains possessed two Opp systems, hereafter named *opp*-I and *opp*-II. The phylogenic relations between *opp* operons within the same species are complex. It has been suggested that in the Hominis group the *opp* operons underwent duplication and divergence. In *M. gallisepticum*, one of the *opp* operons was proposed to be horizontally acquired from a member of the Hominis group [36]. The correct annotation of *oppA* sequences in *Mycoplasma* spp. genomes is problematic because the gene is often identified only as a lipoprotein [39]. In this study, we found a putative *oppA* gene in the MS-H *opp*-I cluster, based on the 28% similarity of the product to *M. canadense oppA*. Unlike *M. gallisepticum*, where the two operons are found in tandem, the *M. synoviae opp*-I and *opp*-II systems were distant from each other within the chromosome. Moreover, the order of the *opp* genes differed between the two operons in MS-H and WVU-1853, as has been previously noted for MS53 [39]. Homologous proteins encoded by the two gene clusters share low sequence similarity, possibly indicating that they are involved in the acquisition of different nutrients. The *M. synoviae opp*-I operon *(*Fig. 4a) is similar to the *opp*-1 subtype of *Staphylococcus aureus* [40] but contains 2 pseudogenes, *oppF* in MS-H and *oppA* in WVU-1853. In WVU-1853, a premature stop codon in *oppA* was close to 3′ end of the gene while the frameshift mutation in MS-H *oppF* allows only the translation of 157 of 797 amino acids, most likely resulting in loss of function of the protein. In contrast, the *opp*-II operon (Fig. 4b) is intact and identically organised in all three strains, but differs from other avian mycoplasma species with respect to its gene arrangement [39]. Co-existing *opp* systems with different, but partially redundant, substrate specificity have been described in *Bacillus subtilis*, in which the inactivation of one system may be compensated for by the presence of an active second operon [41, 42]. It is tempting to speculate that in WVU-1853 the *oppA* pseudogene from *opp*-I is at least partially complemented by the full *oppA* copy from the *opp*-II operon, while in MS-H the pseudogenisation of *oppF* in *opp*-I is not compensated by the *opp*-II copy, which has resulted in attenuation of virulence. Experimental verification of this hypothesis is required to establish whether the two OppA transporters can co-operate or display broad substrate specificity, whereas OppF and other components of the Opp system have more exacting interactions in *M. synoviae*. Alternatively, as has been hypothesized for OppD1 in *M. gallisepticum* [36], it is possible that the two OppF proteins form dimers in the transport complex and the product of the partial copy of *oppF*-I in MS-H may retain the capacity to form a heterodimer with the product of full-length copy of *oppF*-II, resulting in a functional reduction thus a decrease in virulence.

Other transport systems operons were also found to contain pseudogenes. Advanced pseudogenisation was noted for the *sgaABT* gene cluster, which is predicted to encode an ascorbate-specific PTS and which is located next to a strain-variable region containing Type I and Type III RM systems, as well as an integrase gene (see Fig. 3d). Sequences encoding the subunits EIIC (*sgaT*-2), IIB (*sgaB*) and IIA (*sgaA*) appeared to be apparently intact in MS53, but various degrees of pseudogenisation were observed in the 2 other strains. In MS-H and WVU-1853, *sgaT*-2 was split into four fragments. In MS-H, *sgaB* contained a frameshift very close to the start codon, but this gene was intact in MS53 and WVU-1853. The integrity of the PTS transporter locus in MS53 might be correlated with the absence of a RM system and associated sequences in its vicinity (see Fig. 3d). As with the *opp*-I and *opp*-II operons, a second ascorbate-specific PTS locus containing intact sequences for the transport subunits IIA, IIB and EIIC was identified in the genome of all 3 *M. synoviae* strains. This second locus may compensate for the pseudogenisation seen in MS-H and WVU-1853. Multiple copies of the ascorbate-specific PTS are often seen in bacteria [43], and are involved in acquisition of L-ascorbate by the organism under anaerobic conditions [44, 45]. However, orphan IIA and/or IIB and/or IIC homologues of these systems are also often found, and may be residues of genomic minimisation [46]. In MS-H, the *rpiR*-like transcriptional regulator gene, lying downstream of the *nanA* variant and the IS1634, was also split into two pseudogenes, but was intact in MS53 and WVU-1853 (Fig. 3c). It is known that NanA is involved in the breakdown and utilisation of sialic acid [47], and RpiR belongs to a family of transcriptional regulators, some of which are able to repress or activate the expression of *nan* genes [48–51]. As transcription regulatory systems in mycoplasmas are not well understood, it is not possible to predict whether the pseudogenisation of *rpiR* gene might modify sialic acid metabolism in *M. synoviae*. In addition, restriction modification sequences are often present as pseudogenes in bacterial genomes. In MS-H, these pseudogenes affect a type I restriction-modification specificity subunit S and a type III restriction-modification system methylation subunit.

### Clustered regularly interspersed palindromic repeats and evidence of horizontal gene transfer

**Fig. 5:**
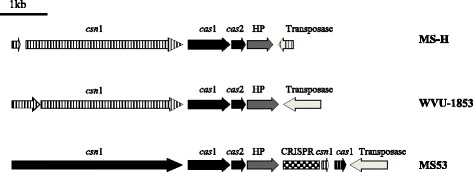
Organisation of the CRISPR/Cas system genes in the 3 *M. synoviae* genomes. Solid arrows represent genes. Vertically hatched arrows represent pseudogenes. The checked rectangle indicates the CRISPR repeats

All 3 *M. synoviae* strains harbor a typical CRISPR-associated Cas system, with *csn1* sequentially followed by *cas1*, *cas2* and a putative *csn2* in a contiguous operon (Fig. 5). Homologs of these proteins are also encoded in the genomes of other *Mycoplasma* species, including *M. ovipneumoniae, M. arthritidis*, *M. hyosynoviae* and *M. gallisepticum* [52, 53]. The operon structure was consistent with a type II (Nmeni subtype) CRISPR/Cas system [52, 54, 55]. However, MS53 was the only *M. synoviae* strain found to possess a CRISPR array, formed by 11 spacers separated by 36 -bp repeat units, suggesting a record of past foreign DNA invasions in this strain. Small duplicated fragments of *csn1* and *cas1* were positioned downstream of the CRISPR array (Fig. 5). Among the CRISPR- associated genes, *csn1* appeared intact in MS53, but was present as a pseudogene in WVU-1853 and MS-H. In WVU-1853, *csn1* was disrupted by a single nucleotide deletion resulting in a frameshift [11]. In MS-H, *csn1* was disrupted by an 11 nucleotide insertion. In both strains these mutations were positioned within the 5′ region of the coding sequence and were therefore likely to result in the loss of CRISPR function, as has been observed previously in *Streptococcus thermophilus* [56]. An IS element was found downstream of the CRISPR -associated genes in all three strains. Genome alignments of all 3 *M. synoviae* strains with *M. gallisepticum*, another mycoplasma species infecting chickens, detected 11 regions sharing similarity of more than 90%, suggesting that some horizontal gene transfer has occurred between these two species. The majority of these regions have been previously reported as putatively transferred [10], and notably include the *vlhA* haemagglutinin genes [57, 58]. In addition, the deoxyribose-phosphate aldolase (*deoC*) sequence fragment (MSH_03520), which was located upstream of an IS1634 in MS-H (Fig. 3a) and was not found in WVU-1853 and MS53, was very similar to a sequence in *Mycoplasma gallisepticum* strain R, sharing 94% amino acid similarity with it. In *Mycoplasma gallisepticum* strain R this sequence is also flanked by IS-related sequences. Moreover, while MS53 and WVU-1853 have only one ATP-binding helicase CDS, a second helicase gene was found in MS-H (MSH_07190) (Table 3). This second copy, flanked by two IS30 family transposase genes (Fig. 3b), is also detected in other *Mycoplasma* species, including *M. gallinaceum* strain B2096-8B, with which it had 93% amino acid similarity. *M. gallinaceum* is a mycoplasma of low pathogenicity that shares the same habitat [59, 60] and phylogenic group [61] as *M. synoviae*. Additionally, two siderophore-mediated iron transport proteins were also identified as putatively horizontally transferred in a single *M. synoviae* genome region (536617–551,599 in MS-H). Siderophores are specific Fe (III)-binding agents produced by many microorganisms that mediate iron scavenging from the environment of the host [62]. Siderophore-mediated iron transport system related genes are considered as virulence factors for many bacterial pathogens because of their critical role in adaption to iron-limited conditions within the host [63, 64]. Although the iron-acquisition mechanisms in mycoplasmas remain largely unknown [65], this putative siderophore-associated iron transport system raises the possibilities that siderophores may contribute to the pathogenesis of infection with *M. synoviae*.

## Conclusion

The MS-H live vaccine has been used to prevent infection with virulent *M. synoviae* in poultry industry for more than two decades and is now used in many countries around the world. As a result it is possible that MS-H is the most prevalent strain of *M. synoviae* globally. Comparative genome analyses revealed a number of features unique to MS-H, WVU-1853 or MS53, but the genomes of these three strains were largely similar to each other. In particular, striking similarities were found between strains MS-H and WVU-1853, despite their distinct geographical origins and dates of isolation. This apparently low genome variability within the species supports the evidence from the field that MS-H protects against *M. synoviae* in a wide range of countries. Differentiation of vaccine strains from field strains is always challenging because of the limited number of genetic markers available for development of routine tests. In this study, we identified genetic features unique to the MS-H vaccine strain, that may be able to be targeted in the future for diagnostic purpose. Strain-variable sequences, putatively acquired by horizontal transfer, and pseudogenes play a critical role in the genomic plasticity of *M. synoviae*. This is exemplified by the discovery of a large inversion in the vaccine strain MS-H adjacent to a short region potentially transferred from *M. gallisepticum*, and an ATP-binding helicase gene putatively acquired from *M. gallinaceum.* Although the 3 *M. synoviae* strains had similar numbers of pseudogenes, the slightly more advanced pseudogenisation of MS-H could be explained by the chemical mutagenesis used to produce the vaccine. It is not yet clear whether the pseudogenisation of some of these genes is associated with attenuation of virulence that is characteristic of the MS-H vaccine. The complete genomic sequencing of MS-H is the first step towards a thorough comparison with its parental strain 86,079/7NS, currently underway in our laboratory, that will help address this question.

## Additional files

Additional file 1: Table S1. Composition of the inverted region in MS-H. (DOCX 23 kb)

Additional file 2: Table S2. Nucleotide positions of the unique gene locus tags in MS-H genome. (DOCX 21 kb)

## Abbreviations

CRISPR: Clustered Regularly Interspersed Short Palindromic Repeats
DeoC: Deoxyribose-phosphate aldolase
GI: Genomic Island CDS: Coding Sequence
IS: Insertion Sequences
LicA: Lichenan-specific IIA component
NanA: Acetylneuraminate lyase
NCBI: National Centre for Biotechnology Information
NGS: Next Generation Sequencing
ORF: Open Reading Frame
PHAST: PHAge Search Tool
PTS: Phosphotransferase system
RM system: Restriction Modification system
Opp: Oligopeptide permease
