# Supplementary material for: Genome analysis of Mycoplasma synoviae strain MS-H, the most common M. synoviae strain with a worldwide distribution
Source: BMC Genomics. 2018 Feb 2;19:117. doi: 10.1186/s12864-018-4501-8 (PMC5797395; doi:10.1186/s12864-018-4501-8)
Supplement: Supplementary file 1 — Composition of the inverted region in MS-H. (DOCX 23 kb) [file 12864_2018_4501_MOESM1_ESM.docx]

Table S1. Composition of the inverted region in MS-H

| **Locus tag** | **Gene start** | **Gene end** | **Strand** | **Product** |
| --- | --- | --- | --- | --- |
| MSH_03130 | 321756 | 321829 | -1 | tRNA-Gly |
| MSH_03140 | 322042 | 422893 | -1 | DegV-like protein |
| MSH_03150 | 322976 | 324418 | 1 | Hypothetical protein |
| MSH_03160 | 324427 | 324897 | 1 | Transcription termination protein NusB |
| MSH_03170 | 324869 | 325369 | 1 | Hypothetical protein |
| MSH_03180 | 325373 | 326536 | 1 | Cysteine desulfurase, SufS subfamily |
| MSH_03190 | 326523 | 326951 | 1 | Putative iron-sulfur cluster assembly scaffold protein for SUF system, SufE2 |
| MSH_03200 | 326938 | 328179 | 1 | DNA polymerase IV |
| MSH_03210 | 328181 | 329266 | 1 | Nicotinate-nucleotide adenylyltransferase), YqeK |
| MSH_03220 | 329266 | 329873 | 1 | Ribosomal large subunit pseudouridine synthase B |
| MSH_03230 | 330083 | 332680 | 1 | DNA gyrase subunit A |
| MSH_03240 | 332752 | 334923 | -1 | Hypothetical protein |
| MSH_03250 | 335203 | 335685 | -1 | Hypothetical protein |
| MSH_03260 | 335660 | 335923 | -1 | Hypothetical protein |
| MSH_03270 | 336106 | 336756 | -1 | Hypothetical protein |
| MSH_03280 | 337459 | 340131 | -1 | ATP-dependent protease La Type I |
| MSH_03290 | 340172 | 342469 | -1 | Leucyl-tRNA synthetase |
| MSH_03300 | 342478 | 343248 | -1 | Putative membrane integrated oxidoreductase |
| MSH_03310 | 343248 | 343868 | -1 | Uracil phosphoribosyltransferase |
| MSH_03320 | 344026 | 344967 | 1 | Phenylalanyl-tRNA synthetase alpha chain |
| MSH_03330 | 344971 | 345645 | 1 | Uracil-DNA glycosylase, family 1 |
| MSH_03340 | 345629 | 347809 | 1 | Phenylalanyl-tRNA synthetase beta chain |
| MSH_03350 | 347812 | 349077 | 1 | Serine hydroxymethyltransferase |
| MSH_03360 | 349105 | 349401 | -1 | hypothetical protein |
| MSH_03370 | 349403 | 350959 | -1 | Methionyl-tRNA synthetase |
| MSH_03380 | 350961 | 351752 | -1 | tRNA (adenine37-N(6))-methyltransferase TrmN6 |
| MSH_03390 | 351727 | 353934 | -1 | 3'-to-5' exoribonuclease RNase R |
| MSH_03400 | 354021 | 354440 | -1 | Ferroxidase |
| MSH_03410 | 354464 | 355546 | -1 | Endo-1,4-beta-glucanase |
| MSH_03420 | 355673 | 358171 | 1 | Valyl-tRNA synthetase |
| MSH_03430 | 358159 | 358995 | 1 | Methenyltetrahydrofolate cyclohydrolase |
| MSH_03440 | 358995 | 359972 | 1 | 6-phosphofructokinase |
| MSH_03450 | 360049 | 360627 | -1 | Mobile element protein |
| MSH_03460 | 360729 | 361808 | -1 | Mobile element protein |
| MSH_03470 | 362172 | 364103 | -1 | Hypothetical protein |
| MSH_03480 | 364087 | 364422 | -1 | Hypothetical protein |
| MSH_03490 | 364859 | 365713 | -1 | Hypothetical protein |
| MSH_03500 | 365717 | 366730 | -1 | Asparaginyl-tRNA synthetase-related protein |
| MSH_03510 | 367191 | 367358 | 1 | IS30 element |
