# Supplementary material for: Genome analysis of Mycoplasma synoviae strain MS-H, the most common M. synoviae strain with a worldwide distribution
Source: BMC Genomics. 2018 Feb 2;19:117. doi: 10.1186/s12864-018-4501-8 (PMC5797395; doi:10.1186/s12864-018-4501-8)
Supplement: Supplementary file 2 — Nucleotide positions of the unique gene locus tags in MS-H genome. (DOCX 21 kb) [file 12864_2018_4501_MOESM2_ESM.docx]

Table S2. Nucleotide positions of the unique gene locus tags in MS-H genome

| **Locus tag** | **Nucleotide position** | **Gene product** |
| --- | --- | --- |
| MSH_00300 | 28019 -> 28492 | N-acetylneuraminate lyase variant |
| MSH_00310 | 28955 -> 28536 | Transcriptional regulator |
| MSH_00330 | 29383 -> 29156 | Transcriptional regulator |
| MSH_00500 | 43386 -> 42931 | Translation elongation factor G |
| MSH_00560 | 50164 -> 49772 | Choline kinase family |
| MSH_01200 | 107067 -> 106285 | Multiple sugar ABC transporter, ATP-binding protein |
| MSH_01840 | 175663 -> 173771 | Hypothetical protein |
| MSH_01930 | 184582 -> 183302 | Hypothetical protein |
| MSH_01960 | 186751 -> 186948 | Hypothetical protein |
| MSH_01970 | 188503 -> 187223 | Hypothetical protein |
| MSH_02140 | 207173 -> 206346 | Type II restriction enzyme MjaIII |
| MSH_02150 | 208962 -> 207925 | Methyl-directed repair DNA adenine methylase |
| MSH_02310 | 221997 -> 227897 | Hypothetical protein |
| MSH_02940 | 305844 -> 305725 | 3'-to-5' oligoribonuclease A |
| MSH_02950 | 306074 -> 306421 | 3'-to-5' oligoribonuclease A |
| MSH_02960 | 306479 -> 307435 | 3'-to-5' oligoribonuclease A |
| MSH_02990 | 308913 -> 309719 | Aspartate-ammonia ligase |
| MSH_03480 | 364422 -> 364087 | Hypothetical protein |
| MSH_03490 | 365713 -> 364859 | Hypothetical protein |
| MSH_03500 | 366730 -> 365717 | Asparaginyl-tRNA synthetase-related protein |
| MSH_03520 | 368566 -> 368291 | Deoxyribose-phosphate aldolase |
| MSH_03740 | 397534 -> 395855 | Oligopeptide transport ATP-binding protein OppF |
| MSH_03750 | 398244 -> 397774 | Oligopeptide transport ATP-binding protein OppF |
| MSH_03790 | 404758 -> 401783 | Lipoprotein |
| MSH_04020 | 423554 -> 427795 | Hypothetical protein |
| MSH_04620 | 489742 -> 487808 | Hypothetical protein |
| MSH_04790 | 507373 -> 507642 | Hypothetical protein |
| MSH_04880 | 513817 -> 514188 | Hypothetical protein |
| MSH_04960 | 522769 -> 534519 | Hypothetical protein |
| MSH_05040 | 538680 -> 536617 | Siderophore-mediated iron transport protein |
| MSH_05850 | 637916 -> 638287 | Hypothetical protein |
| MSH_05860 | 638473 -> 638802 | Hypothetical protein |
| MSH_05940 | 649545 -> 648349 | Hypothetical protein |
| MSH_06430 | 686373 -> 686516 | CRISPR-associated protein, Csn1 |
| MSH_06440 | 686627 -> 690139 | CRISPR-associated protein, Csn1 |
| MSH_07180 | 762003 -> 760981 | IS30 family transposase |
| MSH_07210 | 766383 -> 765667 | IS30 family transposase |
| MSH_07190 | 765087 -> 762259 | ATP-dependent DNA helicase |
| MSH_07360 | 780322 -> 780450 | Hypothetical protein |
| MSH_07370 | 780478 -> 780834 | Hypothetical protein |
| MSH_07380 | 780812 -> 781477 | ABC transporter ATP-binding and permease protein |
| MSH_07610 | 809290 -> 808670 | Hypothetical protein |
| MSH_07640 | 810593 -> 810363 | PTS system enzyme IIB component |
| MSH_07650 | 811270 -> 810704 | Ascorbate-specific PTS system, EIIC component |
| MSH_07660 | 811730 -> 811542 | Ascorbate-specific PTS system, EIIC component |
| MSH_07670 | 811882 -> 811763 | Ascorbate-specific PTS system, EIIC component |
| MSH_07680 | 812098 -> 811931 | Ascorbate-specific PTS system, EIIC component |
| MSH_07490 | 795353 -> 795622 | Type III restriction-modification system methylation subunit |
| MSH_07500 | 795853 -> 796308 | Type III restriction-modification system methylation subunit |
| MSH_07510 | 796998 -> 797807 | Hypothetical protein |
| MSH_07520 | 797795 -> 798205 | Hypothetical protein |
| MSH_07530 | 798227 -> 798664 | Hypothetical protein |

Table S2 - Continued

| MSH_07540 | 798775 -> 799170 | Hypothetical protein |
| --- | --- | --- |
| MSH_07560 | 800370 -> 799453 | Integrase |
| MSH_07570 | 801887 -> 800385 | Type I restriction-modification system, specificity subunit S |
| MSH_07580 | 802665 -> 802075 | Type I restriction-modification system, specificity subunit S |
| MSH_07590 | 805898 -> 802701 | Type I restriction-modification system, restriction subunit R |
| MSH_07600 | 808458 -> 805906 | Type I restriction-modification system, DNA-methyltransferase subunit M |
